# Role of Vitamin D in Parkinson's Disease

**DOI:** 10.5402/2012/134289

**Published:** 2012-03-07

**Authors:** Khanh Lương, Lan Nguyễn

**Affiliations:** Vietnamese American Medical Research Foundation, Westminster, CA 92683, USA

## Abstract

Parkinson's disease (PD) is the second most common form of neurodegeneration in the elderly population. Clinically, it is characterized by tremor, rigidity, slowness of movement, and postural imbalance. A significant association between low serum vitamin D and PD has been demonstrated, suggesting that elevated vitamin D levels might provide protection against PD. Genetic studies have helped identify a number of proteins linking vitamin D to PD pathology, including the major histocompatibility complex (MHC) class II, the vitamin D receptor (VDR), cytochrome P450 2D6 (CYP2D6), chromosome 22, the renin-angiotensin system (RAS), heme oxygenase-1 (HO-1), poly(ADP-ribose) polymerase-1 gene (*PARP-1*), neurotrophic factor (NTF), and Sp1 transcription factor. Vitamin D has also been implicated in PD through its effects on L-type voltage-sensitive calcium channels (L-VSCC), nerve growth factor (NGF), matrix metalloproteinases (MMPs), prostaglandins (PGs) and cyclooxygenase-2 (COX-2), reactive oxygen species (ROS), and nitric oxide synthase (NOS). A growing body of evidence suggests that vitamin D supplementation may be beneficial for PD patients. Among the different forms of vitamin D, calcitriol (1,25-dihydroxyvitamin D_3_) is best indicated for PD, because it is a highly active vitamin D_3_ metabolite with an appropriate receptor in the central nervous system (CNS).

## 1. Introduction

Parkinson's disease (PD) is a movement disorder characterized by tremor, rigidity, slowness of movement, and postural imbalance. There is evidence of abnormalities in the vitamin D-endocrine system in PD patients, including low bone mineral density (BMD), decreased vitamin D levels, and increased bone turnover makers (bone alkaline phosphatase and urinary N-terminal telopeptide of type I collagen) compared to controls [1]. These factors, combined with balance problems, are the probable reasons for the high incidence of fractures, especially of the hip, reported in elderly women with PD [2]. Sunlight exposure can increase the BMD of PD by increasing serum 25-hydroxyvitamin D_3_ (25OHD) levels [3]. In another study, serum 25OHD and BMD were reported to be reduced in PD patients. The BMD Z-score of the trochanter was directly correlated to the degree of physical activity and total body BMD Z-score correlated to the degree of rigidity [4]. Osteoporosis and osteopenia are common findings in PD patients, affecting up to 91% of women and 61% of men [5]. Decreased BMD, low concentrations of serum ionized calcium, and compensatory hyperparathyroidism increase the risk of hip fracture in PD patients [1]. Despite an abundance of correlational studies, it is unknown whether vitamin D deficiency is a cause or consequence of PD. In the present paper, we review the hypothesized roles of vitamin D in PD pathogenesis.

## 2. Genomic Factors Associated with Vitamin D in Parkinson's Disease

### 2.1. Major Histocompatibility Complex (MHC)

Studies have suggested that several genes in the MHC region promote susceptibility to PD. Significantly increased levels of MHC class II expressions were detected in the cerebrospinal fluid (CSF) monocytes of PD patients [6]. Human leukocyte antigen (HLA) genes have also been implicated in PD, and large numbers of HLA-DR-positive reactive microglia were detected in the substantia nigra (SN) and the nigrostriatal tract in PD patients [7, 8]. HLA-DR-positive microglia have also been found in these regions in a case of Parkinson's-associated dementia in Guam [8]. Conversely, calcitriol (1,25-dihydroxyvitamin D_3_) is known to stimulate phagocytosis but suppress MHC class II antigen expression in human mononuclear phagocytes [9, 10]. Calcitriol also decreases interferon-gamma-induced HLA-DR antigen expression on normal and transformed human keratinocytes [11, 12]. These findings suggested that vitamin D may modulate MHC class II antigen expression in PD.

### 2.2. Vitamin D Receptor (VDR)

There is ample evidence for vitamin D involvement in mammalian brain function. VDR and 1*α*-hydroxylase, the enzyme responsible for the formation of active vitamin D in the human brain, are found in the large neurons of the SN, as well as in neurons and glial cells in the hypothalamus [13]. VDR, a nuclear receptor, is restricted to the nucleus but 1*α*-hydroxylase is distributed throughout the cytoplasm. The presence of these proteins in the CNS suggests that vitamin D is active within the brain. VDR knockout mice have muscular and motor impairment [14]. Genetic studies provide an opportunity to link molecular variations with epidemiological data, DNA sequence variations, such as polymorphisms, exert subtle biological effects on the expression and functionality of proteins. VDR mRNA was identified as a potential blood marker for PD [15]. In a Korean population, the VDR BsmI genotype is reported to be associated with PD [16]. Butler et al. [17] reported that the VDR gene is a potential susceptibility gene for PD in the Caucasian population. These reports suggested a role of vitamin D in PD.

### 2.3. The Cytochrome P_450_ (CYP)

CYP superfamily of enzymes is responsible for the oxidation, peroxidation, and/or reduction of vitamins, steroids, and xenobiotics, as well as the metabolism of drugs. CYP2D6, an important member of this superfamily, is expressed in neurons in the brain and gut and may be influenced by polymorphic expression. There is a higher prevalence of the poor-metabolizing CYP2D6*4 allele in PD patients compared with controls (20.7% versus 11.0%) [18]. In case-control and meta-analysis studies, the CYP2D polymorphism was found to be associated with PD [19, 20]. Other studies, however, did not find an association between the CYP2D6 polymorphism and PD in an Asian population [21, 22]. Although the poor metabolizer genotype has a small but highly significant association with PD, it would be easily missed in studies with modest numbers of subjects. CYP2D6 protein and enzymatic activity are completely absent in less than 1% of Asian people and in up to 10% of Caucasians with two null alleles [23]. Singh et al. reported the expression of CYP2D22, an ortholog of human CYP2D6, in mouse striatum and its modulation in MPTP-induced PD phenotype and nicotine-mediated neuroprotection [24]. CYP2D6 is a potential 25-hydroxylase, which converts vitamin D_3_ into 25OHD, and vitamin D 25-hydroxylase deficiency resulted in vitamin D deficiency [25]. Moreover, the CYP2D and PD loci are located on the same chromosome 22 [26, 27]. Deletion of chromosome 22q11 syndrome was reported to be associated with PD [28, 29]. Interestingly, patients with a deletion of chromosome 22q11 showed a reduced BMD, serum calcium, and PTH levels; 11% and 8% of these patients had serum 25OHD levels under 20 ng/ml and abnormal serum 1,25OHD levels, respectively [30].

### 2.4. The Renin-Angiotensin System (RAS)

The primary function of the RAS is to maintain fluid homeostasis and regulate blood pressure. Several components of the RAS and its receptors are found in the CNS [31–34], suggesting that RAS is important in the brain. CSF levels of angiotensin-converting enzyme (ACE) activity were reported to be decreased in PD patients and increased with dopaminergic treatment [35, 36]. In addition, the ACE inhibitor perindopril has been shown to exert beneficial effects on the dopaminergic system [37, 38]. After four weeks of treatment with perindopril, patients with PD had faster improvement in motor response after L-dopa and a reduction in “on phase” peak dyskinesia [39]. The frequency of the homozygous DD genotype of the ACE gene was significantly increased in patients with PD, and is also higher in PD patients with L-dopa-induced psychosis versus without psychosis [40, 41]. However, other studies did not reveal any associations between ACE polymorphisms, PD, and of L-dopa-induced adverse effects [42, 43]. The dissimilar findings may be attributable to differences between Chinese and Caucasian populations. Interestingly, there is also an interaction between vitamin D and the RAS. The use of ACE inhibitors by the ACE DD genotype has been shown to decrease the level of calcitriol [44]. In addition, genetic disruption of the VDR resulted in overstimulation of the RAS with increased production of renin and angiotensin II, thereby leading to high blood pressure and cardiac hypertrophy. Treatment with captopril reduced cardiac hypertrophy in VDR knockout mice [45], suggesting that calcitriol may function as an endocrine suppressor of renin biosynthesis. Vitamin D has also been reported to decrease ACE activity in bovine endothelial cells [46]. The findings suggested that vitamin D might affect ACE activity in PD. 

### 2.5. Heme Oxygenase-1 (HO-1)

HO-1 is a stress protein that may confer cytoprotection by enhancing catabolism of pro-oxidant heme to the radical scavenging bile pigments, biliverdin, and bilirubin. The HO-1 gene can be upregulated by a host of noxious stimuli and is induced in CNS tissues affected by neurological diseases [47]. In the normal brain, basal HO-1 expression is low and restricted to small groups of scattered neurons and neuroglia [48]. In the brains of PD patients, the HO-1 is highly overexpressed in astrocytes within the SN and in Lewy bodies found in affected dopaminergic neurons [49]. Serum HO-1 levels are increased in PD patients but not in patients with Alzheimer's disease (AD) [50], suggesting a systemic antioxidant reaction to a chronic oxidative stress state that is unique to PD. Similarly, calcitriol delayed of HO-1 immunoreactivity after the postlesional survival time of 12 hours concomitant with a reduction in glial fibrillary acidic protein immunoreactivity in remote cortical regions affected by a secondary spread of injury in glial cells of the focal cerebral ischemic [51], thereby supporting the protective role of calcitriol in postcellular injury.

### 2.6. Poly(ADP-Ribose) Polymerase-1 (PARP-1)

PARP-1 is a nuclear protein that can promote either neuronal death or survival under certain stress conditions. Overexpression of PARP-1 has been reported in the dopaminergic neurons of the SN in PD [52]. PARP-1 is also implicated in 1-methyl-4-phenyl-1,2,3,6-tetrahydropyridine- (MPTP-) induced neurotoxicity *in vivo* [53]. MPTP is a neurotoxin that induces parkinsonian symptoms in human and animals, but mice lacking PARP gene are spared from MPTP neurotoxicity [54]. Therefore, PARP inhibitors have proved to be valuable tools in PD model [55, 56]. PARP-1 variants are reported to be protective against PD [57]. In addition, increased levels of vitamin D seem to downregulate PARP-1 expression; PARP-1 levels decrease following calcitriol treatment in NB4 cells, which represent an acute promyelocytic leukemia cells [58]. Vitamin D exerts a concentration-dependent inhibitory effect on PARP-1 in the human keratinocyte cells [59]. Vitamin D-induced downregulation of PARP is further enhanced by nicotinamide in human myeloblastic leukemia cells [60]. Furthermore, PARP is attenuated in the hippocampus of rats that received dexamethasone and vitamin D [61], suggesting that the anti-inflammatory effects of dexamethasone and vitamin D derives from their ability to downregulate microglial activation. These findings suggested that vitamin D may have a protective role in PD by downregulating PARP.

### 2.7. Neurotrophic Factors (NTFs)

NTFs are keys to surviving various neuronal insults and promote neuronal regeneration following injury [62]. NTFs can exert protective or even restorative effects on PD animal models and dopaminergic cell cultures; key examples include brain-derived neurotrophic factor (BDNF) [63], glial-derived neurotrophic factor (GDNF) [64], mesencephalic-astrocyte-derived neurotrophic factor (MANF) [65], and cerebral dopamine neurotropic factor (CDNF) [66]. Receptors for these NTFs are expressed in the striatum and SN [67, 68]. NTF expression is reduced in the SN of patients with PD [69–71]. Moreover, the C allele of an inotropic CDNF single nucleotide polymorphism (rs7094179) has been suggested to infer susceptibility to PD in a Korean population [72], and A allele of BDNF is associated with PD in a Chinese Han population [73]. Interestingly, calcitriol regulates the expression of the low-affinity neurotrophic receptor [74] and increases striatal GDNF mRNA and protein expression in adult rats [75]. *In vivo*, calcitriol is also a potent inducer of GDNF in rat glioma cells [76]. The brains of offspring from vitamin D-deficient dams are characterized by diminished expression of neurotrophic factors [77]. Furthermore, calcitriol protects against dopamine loss from 6-hydroxydopamine- (6-OHDA-) lesioned rats by increasing GDNF and partially restores tyrosine hydroxylase expression in SN and striatum [78, 79].

### 2.8. Sp1 Transcription Factor

Sp1 transcription factor is a member of an extended family of DNA-binding proteins that is acetylated in response to oxidative stress in neurons [80]. The Sp1 family of proteins plays an important role in controlling the expression of the dopamine transporter gene within dopaminergic neurons [81] and also regulates expression of rat dopamine receptor gene [82]. On the other hand, binding sites for the transcription factor Sp1 have been implicated in the transcription of several genes by hormones. In cultured human fibroblasts, the level of CYP24 (25-OHD 24-hydroxylase) mRNA plays a key role in the metabolism of 1,25OHD and increases up to 20,000-fold in response to calcitriol. Two vitamin D-responsive elements (VDREs) located upstream of the CYP24 gene are primarily responsible for the increased mRNA levels, and Sp1 acted synergistically with these VDREs for the induction [72]. The mVDR promoter is controlled by Sp1 sites [73] and functions as the transactivation component of the VDR/Sp1 complex to trigger gene expression [74]. Moreover, the genes encoding Sp1 and VDR were mapped to human chromosome 12q [75, 76].

## 3. Nongenomic Role of Vitamin D in Parkinson's Disease

### 3.1. Diabetes Mellitus (DM)

Glucose is the molecule necessary to produce energy in the brain. A link between DM and PD has been suggested in several reports, but the results have been inconsistent. Insulin receptors and their mRNAs are localized in the SN [83, 84]. A high incidence of abnormal glucose tolerance has been reported in PD and seems to be exacerbated by L-dopa treatment [85]. DM has been associated with the risk of developing PD [86, 87] whereas others reported from protective to no associations with PD [88–90]. Human and experimental animal studies, however, demonstrated neurodegeneration associated with peripheral insulin resistance [91]. In a 6-OHDA model of PD, striatal insulin resistance was observed in the striatum [92], and patients with PD exhibited increased autoimmune reactivity to insulin [93]. Individuals newly diagnosed with PD display reduced insulin-mediated glucose uptake [94], which is hypothesized to be due to inhibit early insulin secretion and hyperglycemia after glucose loading [95]. Furthermore, chronic hyperglycemia decreased limbic extracellular dopamine concentrations and striatal dopaminergic transmission in streptozotocin-induced diabetic rats [96, 97]. Vitamin D levels may provide a link between the diseases; serum 1,25OHD and 25OHD levels are low in diabetic patients [98, 99], and diabetic rats had an increased metabolic clearance rate of 1,25OHD [100]. Interestingly, a significant high prevalence of vitamin D insufficiency is reported in patients with PD [101, 102]. A significant inverse association between serum vitamin D and PD was demonstrated [103] and suggested that high vitamin D status might provide protection against PD. In diabetic-induced rats, vitamin D and insulin treatment markedly recovered the levels of altered gene (cholinergic, dopaminergic, and insulin receptors) expression and its binding parameters nearly to those of the control rats [104]. Maternal vitamin D deficiency was reported to alter the expression of genes involved in dopamine specification in the developing rat mesencephalon [105]. Calcitriol has been shown to protect dopamine neuronal toxicity induced by 6-OHDA and the combination of L-buthionine sulfoximine and MPTP, thereby restoring motor activity in the lesioned animals [106, 107]. Furthermore, vitamin D was reported to improve rigidity and akinesia and reduce levodopa dosage in a patient with PD [108].

### 3.2. L-Type Voltage-Sensitive Calcium Channels (L-VSCC)

Unlike most neurons in the brain, dopaminergic neurons function as autonomous pacemakers that rely on L-VSCC to generate action potentials at a clock-like 2–4 Hz in the absence of synaptic input [109]. L-VSCC activity during autonomous pacemaking increased the sensitivity of dopaminergic neurons to mitochondrial toxins in an animal model of PD [110]. Epidemiological data supports a link between L-VSCC functioning and the risk of developing PD [111–113]. Pretreatment with the calcium channel antagonist nimodipine has been shown to block the development of MPTP-induced neurotoxicity in animal models [114, 115]. Israpidine, another L-VSCC antagonist, caused a dose-dependent reduction in L-dopa-induced rotational behavior and abnormal involuntary movements in the 6-OHDA-lesioned rat model of PD [116]. With respect to AD, amyloid-*β* protein was reported to promote neurodegeneration by inducing L-VSCC expression and suppressing VDR expression; subsequent treatment with vitamin D protected neurons by preventing cytotoxicity and apoptosis, probably by downregulating L-VSCC and upregulating VDR [117]. Calcitriol decreased L-VSCC activity in aged rats and in neuronal vulnerability with particular impact on reducing age-related changes associated with Ca^2+^ dysregulation [118, 119]. Treatment with 24R, 25 dihydroxyvitamin D_3_ also reduced L-VSCC activity in vascular smooth muscle in rats [120].

### 3.3. Nerve Growth Factor (NGF)

NGF is a small secreted protein that is important for the growth, maintenance, and survival of certain target neurons. NGF has been implicated in maintaining and regulating the septohippocampal pathway, which is involved in learning and memory [121–123]. NGF is also present in the human SN [124] and in the adrenal gland [125]. NGF concentrations are decreased in the SN of the PD and in a rat model of PD [69, 126]. NGF levels showed greater reductions in early states of the disease compared with advanced stages [126], implying that decreased NGF may be involved in the pathogenesis of PD. NGF is reported to protect dopamine neurotoxicity induced by MPTP, rotenone, and 6-OHDA via different pathways [127–129]. The chronic infusion of NGF into the rat striatum resulted in cholinergic hyperinnervation and reduced spontaneous activity of striatal neurons [130]. Moreover, NGF increases survival of dopaminergic grafts, rescues nigral dopaminergic neurons, and restores motor dysfunction in a rat model of PD [131, 132]. In addition, the brains of newborn rats from vitamin D-deficient dams showed reduced expression of NGF [77]. *In vitro*, calcitriol regulated the expression of the VDR gene and stimulated the expression of the NGF gene in Schwann cells [133]. In mouse fibroblasts, calcitriol and vitamin D analogs are reported to enhance NGF induction by increasing AP-1 binding activity to the NGF promoter [134, 135]. These findings suggest a protective role for vitamin D in the CNS.

### 3.4. Matrix Metalloproteinases (MMPs)

MMPs are proteolytic enzymes responsible for extracellular matrix (ECM) remodeling and the regulation of leukocyte migration through the ECM, which is an important step for inflammatory processes. Neuroinflammation is known to contribute significantly to progressive dopaminergic neurodegeneration in PD. MMP involvement has been reported in the degeneration of dopaminergic neurons. MMP-3 expression is increased during lipopolysaccharide- (LPS-) induced dopamine neurotoxicity [136]. MMP-9 is also elevated in MPTP-induced parkinsonism in mice [137]. Application of dopaminergic neurotoxins to two human neuroblastoma cell lines downregulates the transcription and translation of tissue inhibitor of metalloproteinase- (TIMP-) 2 effectively enhancing MMP activity [138]. Exendin-4, which is an analogue of glucagon-like peptide 1 (GLP-1), significantly attenuates the loss of SN neurons and the striatal dopaminergic fibers in the MPTP-induced PD model, and inhibits the expression of MMP-3 [139]. Conversely, the VDR TaqI polymorphism is associated with decreased production of TIMP-1, a natural inhibitor of MMP-9 [140]. Calcitriol modulates tissue MMP expression under experimental conditions [141]. Calcitriol downregulates MMP-9 levels in keratinocytes and may attenuate deleterious effects due to excessive TNF-*α*-induced proteolytic activity associated with cutaneous inflammation [142]. In addition, a vitamin D analogue was reported to reduce the expression of MMP-2, MMP-9, VEGF, and parathyroid hormone-related protein in Lewis lung carcinoma cells [143]. These findings suggested that vitamin D plays a role in modulating MMP activation in PD.

### 3.5. Prostaglandins (PGs)

PGs play a role in inflammatory processes [144]. Cyclooxygenase (COX) participates in the conversion of arachidonic acid into PGs. PGE_2_ is a key product of COX-2 and is increased in the SN of patients with PD and MPTP-induced PD in an animal model [145, 146]. PGE_2_ is also directly and selectively toxic to dopaminergic neurons [147]. PGE_2_ receptors are found on dopaminergic neurons in the rat SN [147]. Overexpression of COX-2 is reported in PD and an MPTP-animal model [148, 149]. COX inhibitors provide neuroprotection in the MPTP-mouse model of PD [150]. Similarly, regular use of COX-2 inhibiting nonsteroidal anti-inflammatory drugs (NSAIDs), such as ibuprofen, has been associated with a decreased incidence of PD [151]. Calcitriol, which reportedly regulates the expression of several key genes involved in PG pathways, decreases PG synthesis [152]. Calcitriol and its analogues have also been shown to inhibit selectively the activity of COX-2 [153]. These findings suggested that vitamin D has a role in anti-inflammatory processes in PD.

### 3.6. Oxidative Stress

Oxidative stress has been suggested to contribute to the pathogenesis of PD. Lymphocytes from untreated PD patients have increase oxidative stress [154]. Analyses of postmortembrains from PD reveal evidence of increased lipid peroxidation in PD SN [155, 156]. A selective superoxide dismutase (SOD) is also increased in the SN of PD patients [157]. Calcitriol administration has been reported to exert a receptor-mediated effect on the secretion of hydrogen peroxide by human monocytes [158]. *In vitro, *monocytes gradually lose their ability to produce superoxide when stimulated; the addition of calcitriol, lipopolysaccharide, or lipoteichoic acid (LTA) restored the ability of stimulated monocytes to produce superoxide and increases the oxidative capacity compared with unstimulated monocytes [159]. Calcitriol can also protect nonmalignant prostate cells from oxidative stress-induced cell death by preventing ROS-induced cellular injuries [160]. Vitamin D metabolites and analogues were reported to induce lipoxygenase mRNA expression, lipoxygenase activity, and ROS production in a human bone cell line [161]. Vitamin D can also reduce lipid peroxidation and induce SOD activity in the rat hepatic antioxidant system [162]. These findings suggested a role of vitamin D in oxidative stress in PD.

### 3.7. Nitric Oxide Synthase (NOS)

NOS is an enzyme involved in the synthesis of nitric oxide (NO), which has also been implicated in PD. In postmortem brains of PD, high levels of NOS expression were found in the nigrostriatal region and basal ganglia [163]. A significant increase in the nitrite content was reported in polymorphonuclear leukocytes of PD patients [164]. Inducible and neuronal NO are increased in both 6-OHDA and MPTP animal models [165, 166]. Moreover, studies with NOS inhibitors and NOS knock-out animals have also confirmed the role of NOS in neurodegeneration [167, 168]. Reduced and oxidized glutathione (GSH) were demonstrated in the SN of patients with PD [169]. Conversely, the activation of 1*α*-hydroxylase in macrophages increases in 1,25OHD, which inhibits inducible NOS (iNOS) expression and reduces NO production by lipopolysaccharide- (LPS-) stimulated macrophages [170]. Thus, calcitriol production by macrophages may provide protection against oxidative injuries caused by the NO burst. Calcitriol is known to inhibit LPS-induced immune activation in human endothelial cells [171]. In experimental allergic encephalomyelitis, calcitriol inhibits the expression of iNOS in the rat CNS [172]. Astrocytes play a pivotal role in CNS detoxification pathways where glutathione (GSH) is involved in the elimination of oxygen and nitrogen reactive species, such as nitric oxide. Calcitriol affects this pathway by enhancing intracellular GSH pools and significantly reduces nitrite production induced by LPS [173].

## 4. Conclusion

 Recent studies have highlighted a possible relationship between vitamin D and PD. Vitamin D may be beneficial in PD patients, as one patient showed improved rigidity and akinesia and was able to decrease their levodopa dosage after vitamin D therapy. Genetic studies have provided opportunities to determine what proteins may link vitamin D to PD pathology. Vitamin D can also act through a number of nongenomic mechanisms, including effects on protein expression, oxidative stress, inflammation, and cellular metabolism. Among the many forms of vitamin D, calcitriol (1,25-dihydroxyvitamin D_3_) is an attractive therapeutic candidate, because it is a particularly active metabolite, and its receptor is expressed in the CNS.
